# Backbone Cyclization of Flavin Mononucleotide-Based Fluorescent Protein Increases Fluorescence and Stability

**DOI:** 10.4014/jmb.2305.05011

**Published:** 2023-08-28

**Authors:** Tingting Lin, Yuanyuan Ge, Qing Gao, Di Zhang, Xiaofeng Chen, Yafang Hu, Jun Fan

**Affiliations:** School of Life Science, Anhui Agricultural University, Hefei, Anhui 230036, P.R. China

**Keywords:** Backbone cyclization, split intein, flavin-based fluorescence protein, linker, stability, anaerobic reporter

## Abstract

Flavin mononucleotide-binding proteins or domains emit cyan-green fluorescence under aerobic and anaerobic conditions, but relatively low fluorescence and less thermostability limit their application as reporters. In this work, we incorporated the codon-optimized fluorescent protein from *Chlamydomonas reinhardtii* with two different linkers independently into the redox-responsive split intein construct, overexpressed the precursors in hyperoxic *Escherichia coli* SHuffle T7 strain, and cyclized the target proteins in vitro in the presence of the reducing agent. Compared with the purified linear protein, the cyclic protein with the short linker displayed enhanced fluorescence. In contrast, cyclized protein with incorporation of the long linker including the myc-tag and human rhinovirus 3C protease cleavable sequence emitted slightly increased fluorescence compared with the protein linearized with the protease cleavage. The cyclic protein with the short linker also exhibited increased thermal stability and exopeptidase resistance. Moreover, induction of the target proteins in an oxygen-deficient culture rendered fluorescent *E. coli* BL21 (DE3) cells brighter than those overexpressing the linear construct. Thus, the cyclic reporter can hopefully be used in certain thermophilic anaerobes.

## Introduction

So far, various fluorescent proteins have been developed for application in biology and biotechnology. Green fluorescence protein (GFP) and its variants, as well as the homologs with different emission and excitation maxima, are being widely applied [1]. However, formation of the fluorophore in GFP family members requires oxygen, which limits their applicability in anaerobes and in anaerobic environments, such as the human gastrointestinal tract. Flavin mononucleotide (FMN)-binding fluorescent proteins, which are derived from either bacterial ﬂmononucleot or a highly conserved family of blue-light plant photoreceptors containing light, oxygen, and voltage (LOV)-sensing domains [1], emit cyan-green fluorescence upon exposure to ultraviolet (UV) or blue light [2]. The LOV-based fluorescent proteins display several advantages over the GFP family members, including function under anaerobic environment, small molecular mass with 110-140 amino acids, and rapid maturation of the fluorophore [3]. The disadvantages of the LOV domains include the relatively low ﬂuorescence quantum yields, color intensities, and less thermostability. Fluorescence from the LOV domain from *C. reinhardtii* (CrLOV) is denatured by incubation at 70°C for 1 h [4]. The selected LOV domain maintains roughly 90% of its initial fluorescence after 24 h incubation at 60°C [5]. By comparison, the engineered GFP variant is extremely stable, retaining nearly 100% fluorescence after incubation at 85°C for 1,000 min [6].

Several approaches are exploited to increase the fluorescence and/or thermostability of the LOV-based reporter [2], including selection of the LOV proteins from various bacterial species [4, 5], mutation of certain amino acids in the LOV domains based on the rational design [7, 8], and direct evolution of certain FMN-based fluorescent proteins [9, 10]. Nonetheless, the use of random mutagenesis to further create brighter protein has not been successful [11]. CrLOV is a photostable, thermostable, and fast-maturing monomeric protein superior to other homologues in brightness and operational pH range [4]. Using the codon-optimized CrLOV reporter, we detected human annexin A1 as the purification tag in *E. coli* for Ca^2+^-dependent phase transition separation [12]. Like that from GFP and red fluorescent protein resistant to sodium dodecyl sulfate (SDS) at low concentration [13], a fluorescent band from CrLOV was observed under UV irradiation after SDS-polyacrylamide gel electrophoresis (SDS-PAGE) was finished [12]. To enhance the CrLOV fluorescence, other strategies are needed.

Backbone cyclization of protein with the desired proximity in N- and C-termini generates covalent constraint, affording the cyclized proteins with improved thermostability, as well as insensitivity to exopeptidase [14]. The split intein constructs with low capacity to cyclize the target proteins in vivo facilitate increased precursor yields. Also, the target proteins are cyclized in vitro under appropriate conditions and cyclic proteins are released from purified precursors [15-17]. The DnaE intein from *Nostoc punctiforme* (Npu DnaE) exhibits ultra-fast splicing reaction, and mutation of ERD residues in the Npu DnaE intein into GEP residues improves the splicing efficiency at the changed amino acid residues surrounding the splice junction [18]. The redox-responsive inteins are designed by ﬂanking the CPG adjacent to the intein’s catalytic cysteine to generate the CPGC motif as the redox trap for reversible formation of the disulfide bond to retard the splicing reaction in hyperoxic *E. coli* cells, and activated in vitro in the presence of the reductant [19]. To investigate this technology application in production of the cyclic protein in vitro, we constructed the redox-trapped split Npu DnaE intein variant, produced the precursor protein in an *E. coli* SHuffle T7 co-expression system for cyclizing the GFP variant in vitro, and identified the cyclic GFP showing the enhanced fluorescence, thermostability, and resistance to exopeptidase digestion [20]. In this study, we used disulﬁde-trap intein technology for cyclizing CrLOV, and confirmed that protein circularization increased the protein fluorescence; however, the effect was related to the incorporated linker. The cyclized CrLOV with the short linker (c-LOV) was more thermostable and resistant to yeast carboxypeptidase Y (CPY) proteolysis than the linear form of CrLOV (l-LOV). Upon induction of the target protein cultured in oxygen-deficient medium at 37°C or 41°C, *E. coli* BL21(DE3) cells overexpressing the precursor for in vivo cyclization of CrLOV displayed higher fluorescence than those producing the linear counterpart.

## Materials and Methods

### Strains, Plasmids and Reagents

*E. coli* strains DH5α and BL21(DE3), and plasmids pET-28b and pET-29b are products of Novagen (USA). *E. coli* Shuffle T7 strain was provided by New England BioLabs (NEB, USA). Reagents for PCR and plasmid construction were bought from Takara (Japan). The pGST-tevS-EmGFP plasmid was constructed for expressing the fusion protein constituted of the His6-tagged glutathione S-transferase (GST), the incorporated tobacco etch virus protease (TEVp) cleavage sequence (tevS, ENLYFQ ↓ G), and green fluorescent protein (GFP) variant emerald GFP (EmGFP). The p28GFP plasmid with sequence coding for the His6-tagged EmGFP inserted into the pET-28b plasmid, the pGST-rpS-eDAL plasmid to express the fusion protein containing the GST tag, human rhinovirus 3C protease (R3P) cleavable sequence (rpS, LEVLFQGP), and the target protein eDAL, the pNpu vector for expressing the split intein variant, the phanA1-CrLOV plasmid for expressing the tagged codon-optimized CrLOV variant, and help vector pBAD33-hPDI were constructed previously [12, 20-22]. The plasmid for expressing the His6-GST tagged R3P was gifted from Professor Jiangye Zang of the University of Science and Technology of China. Primers and gene were synthesized by General Biosystems Co., Ltd. (China). Nickel-nitrilotriacetic acid (Ni-NTA) agarose was bought from Qiagen (Germany). The TEVp variant termed TEVp^5M^ with enhanced protein solubility and folding was purified previously [21]. Purified recombinant yeast CPY with high purity was obtained from Yuanye Bio-Technology Co., Ltd. (China). Reagents for western blotting, mouse monoclonal antibodies for anti-myc and anti-His6 were purchased from Transgen Biotech Corporation (China). The Ultra-15 centrifugal filter tube equipped with the Ultracel-10 membrane is commercially available from Merck-Sigma-Aldrich (USA).

### Construction of the Expression Plasmids

The fragment coding for the CrLOV variant without the stop codon was amplified by PCR using P1 and P2. Amplified products were incubated with NdeI and BamHI, and ligated to the NdeI-BamHI site of the p28Npu to generate the p28NpuLOV plasmid. To ensure that the amino acid sequence of l-LOV was identical with that of c-LOV, the CrLOV coding sequence was amplified using the p28NpuLOV plasmid as the template, and P3 and P4 primers. Considering that a BamHI cut site in the CrLOV coding sequence was inserted into the p28NpuLOV1 plasmid, BglII was introduced as the isocaudarner of BamHI. The amplified products were incubated with BglII and XhoI, and inserted into the BamHI-XhoI site of the pGST-tevS-EmGFP for substitution of the EmGFP coding sequence to express the fusion protein GST-tevS-CrLOV. To compare c-LOV and the linear form of the CrLOV overexpression under different inductions, the amplicon obtained through the use of P1 and P4 for PCR was treated with NdeI and XhoI, and inserted into the NdeI-XhoI site of pET-29b. The fragment encoding CrLOV was fused to the myc tag and rpS was amplified by overlap PCR using P1 and P5, and P1 and P6. The amplified fragment encoding CrLOV-myc was treated with NdeI and BglII, and subcloned into the NdeI-BglII site of the pGST-rpS-eDAL vector to substitute the fragment encoding the GST. Then, the NdeI-BamHI excised fragment coding for CrLOV-myc-rpS was inserted into the NdeI-BamHI site of the p28Npu to yield the plasmid p28NpuLOV1. The BamHI-XhoI excised sequence coding for the EmGFP from the pGST-tevS-EmGFP plasmid was inserted into the same site of the pGST-rpS-eDAL vector for expression of the fusion protein GST-rpS-EmGFP. The primers were listed in Table S1. All inserts were sequenced for identification of the corrections.

### Fluorescence Microscopic Imaging and Flow Cytometric Analysis

The p28NpuLOV plasmids were transformed into *E. coli* BL21(DE3) for overexpression of the precursor undergoing in vivo cyclization of CrLOV. Cells were cultured in LB broth (yeast extract 5 g/l, tryptone 10 g/l, NaCl, 10 g/l, pH 7.5) overnight at 37°C, and diluted to 200-fold. The diluted cells were grown at 37°C until the optical density at 600 nm (OD_600_) reached about 0.5, and the target protein was induced aerobically by using 0.5 mM IPTG at 28°C for 12 h. After centrifugation at 4,000 ×*g* for 10 min at room temperature, collected cells were washed with buffer A (30 mM Tris-HCl, 100 mM NaCl, pH 8.0), and re-suspended with buffer A. Cell fluorescence was monitored with a confocal laser scanning microscope upon exposure of 150 ms (CLSM, Zeiss LSM 800, Jena, Germany). Flow cytometric analysis was conducted on a FACSCalibur Cytometer (Becton, USA) with a 488 nm blue laser and a 510 ± 30 nm bandpass filter. The throughput rate for the analysis was 300 events/s. For each sample, 10,000 events were recorded.

### Overexpression and Purification of the Precursor and In Vitro Cyclization of the CrLOV Variants

The coexpression system was used for increasing productivity of the precursor protein. The p28NpuLOV or p28NpuLOV1 plasmids, mixed with help plasmids pBAD33-hPDI for expressing human protein disulfide isomerase (hPDI), were transformed into *E. coli* SHuffle T7 cells, as described previously [20]. Pre-overexpression of the hPDI was performed with use of 5 mM arabinose until the OD_600_ reached about 0.3 in LB culture (pH 8.0). At OD_600_ about 0.5, the precursor protein was induced by use of 0.5 mM IPTG. Cells were further grown at 37°C for 5 h, harvested by centrifugation at 4,000 ×*g* for 10 min, and re-suspended in buffer B (50 mM sodium phosphate, 300 mM NaCl and 10 mM imidazole, pH 8.0).

The suspended cells were disrupted by sonication on ice, and centrifuged at 12,000 ×*g* for 20 min at 4°C. The soluble fraction was loaded on 4 ml Ni-NTA agarose with pre-equilibrium of buffer B, and washed with 40 ml buffer B containing 30 mM imidazole (pH 8.0). The bound proteins were eluted with buffer B containing 250 mM imidazole (pH 8.0). After the eluent was concentrated with buffer A, Tris (2-carboxyethyl) phosphine (TCEP) was slowly added to the mixture to a final concentration of 4 mM, while the protein concentration was about 1 mg/ml. Then, incubation was carried out at room temperature for 4 h, and the mixture was exchanged with buffer A and loaded onto a Ni-NTA matrix. The His6-tagged proteins were bound to the affinity resin, and the tag-free c-LOV was eluted with buffer A. The eluent was concentrated, and stored at -80°C.

The CrLOV containing the myc-tag and rpS, termed CrLOV1, was also cyclized in vitro, based on the same procedure. The cyclized protein was eluted from the resin with buffer A, and concentrated. The purified CrLOV1 was incubated with purified R3P at a mass ratio of 1: 20 in buffer A. After reaction at 30°C for 8 h, the mixture was subjected to SDS-PAGE and western blot analyses.

### Overexpression, Purification and Tag Removal of the Fusion Protein for the CrLOV

To purify the l-LOV, the His6-GST tagged CrLOV was induced in BL21(DE3) cells in LB culture at 28°C for 12 h, and loaded on Ni-NTA resin (4 ml). The resin was washed with buffer B, and followed by buffer B containing 30 mM imidazole (pH 8.0). On-resin cleavage was finished overnight at about 25°C by using purified TEVp^5M^ with a mass ratio of 15:1. The non-tagged l-LOV was eluted with buffer B and the eluent was exchanged with buffer A. Protein samples were stored at -80°C. The His6-GST tagged R3P was overexpressed and purified using the same procedure.

### Protein Analysis

Protein concentration was analyzed by using the Bradford method, with bovine serum albumin as the standard. The CrLOV fluorescence on the SDS-PAGE gel was recorded [12]. Briefly, protein samples were mixed with the loading buffer and incubated at 37°C for 10 min. After centrifugation, soluble proteins were separated, and the gel was observed under UV light and photographed. To analyze the intact CrLOV after CPY proteolysis, equal amounts of triplicate samples were separated by SDS-PAGE and bands representing the intact proteins were quantified by using the ChemiDoc MP Imaging System (Bio-Rad, USA). To detect the expression level of the EmGFP upon induction under aerobic cultivation or in the oxygen-deficient conditions, western blotting was performed using anti-His6 monoclonal antibody. Protein samples on the gel were transferred to a polyvinylidene fluoride membrane, and immune-blotted with primary antibodies to a dilution of 5,000-fold. For testing the CrLOV1, anti-myc monoclonal antibody was diluted to 5,000-fold. The blotted proteins were treated with horseradish peroxidase-conjugated anti-mouse IgG to a dilution of 5,000-fold. With supply of 4-chloro-1-naphthol solution dissolved in 20% methanol and 0.08% hydrogen peroxide, protein bands on the membrane were photographed.

### Fluorescence Spectra Analysis of Purified c-LOV and l-LOV

Fluorescence spectra from purified c-LOV and l-LOV at the same concentration were scanned using PerkinElmer EnSight (USA). Excitation spectra were recorded by monitoring emission at 540 nm, whereas the excitation wavelength was scanned between 300 and 520 nm. Excitation spectra from 470 nm to 600 nm was scanned with emission at 540 nm while emission spectra were recorded with excitation at 450 nm.

### Thermostability of Purified c-LOV and l-LOV

The solution containing c-LOV was diluted with buffer A to almost equivalent fluorescence with l-LOV at 60°C. Then, purified c-LOV and l-LOV in buffer A were individually incubated for 10 min at every 5°C increment from 60-85°C. The CrLOV fluorescence was measured with emission at 495 [5], using an F-4500 ﬂuorescence spectrometer (Hitachi, Japan). Relative fluorescence for protein samples indicated that the fluorescence signal was divided according to protein amount (mg). The correspondent soluble protein concentrations after heat treatment were measured at A_280_. For SDS-PAGE and fluorescence on SDS-PAGE analyses, almost equal amounts of two soluble LOV species at 60°C for 10 min treatment were used as the reference.

### Hydrolytic Stability of Purified c-LOV and l-LOV

The protein samples with equivalent fluorescence intensities in c-LOV and l-LOV were separately incubated with the CPY at 25°C in buffer C (50 mM PBS buffer, 50 mM NaCl, pH 6.5). From 0 to 10 h and at 2 h increments, relative fluorescence and protein amounts were determined. For SDS-PAGE and fluorescence on SDS-PAGE analyses, almost equal amounts of two CrLOV species before CPY proteolysis were used as the initial reference. The bands representing the intact CrLOV on the SDS-PAGE gel were then calculated.

### Induction of the Fluorescent Protein Reporters Under Low Oxygen Concentration

To detect functional production of the precursor protein or the linear CrLOV construct in *E. coli* BL21(DE3), cells were grown to OD_600_ of 0.6 in 1.5 ml culture. Anaerobic conditions were mimicked, based on cells grown in Eppendorf tubes almost completely filled with LB culture. After IPTG was added at a final concentration of 0.4 mM, the tube was sealed for culturing at 37°C and 41°C for 6 h, respectively. After centrifugation, cells were photographed under UV light. For detecting in vivo cyclization of CrLOV, 0.5 μl CPY was added to the clear lysates containing about 10 μg proteins from cells induced at 37°C, and incubated for 24 h at 25°C. CrLOV fluorescence was displayed on SDS-PAGE gels. The OD_600_ values were measured on a U-2900 UV-vis spectrophotometer (Hitachi, Japan), and relative fluorescence was analyzed. In measuring the relative fluorescence of cells, the fluorescence signal is divided by OD_600_. Five samples under the same induction conditions were measured, and the closest values from three samples were counted.

To further determine anoxic liquid medium in a sealed tube, His6-tagged EmGFP was also induced at 37°C for 5 h. About 1.5 ml cells overexpressing either in 10 ml of LB culture placed in a 50-ml shake flask sealed with breathable film, or in a sealed tube filled with culture, were collected by centrifugation and photographed upon UV light irradiation. The fluorescence at emission 515 nm for the EmGFP was measured. The His6-GFP production was detected by western blotting using anti-His6 antibodies.

### Statistical Analysis

For in vitro analysis, three technical replicates were conducted, and data were calculated. For measuring cell fluorescence, six colonies were picked randomly, and the closest values from three colonies for culturing were calculated. Data were represented as means ± SD, evaluated using a one-tailed *t*-test, and analyzed using SPSS ver. 22 (SPSS Inc., USA).

## Results

### Use of the Split Npu DnaE Variant for Cyclizing the CrLOV

**Fig. 1:**
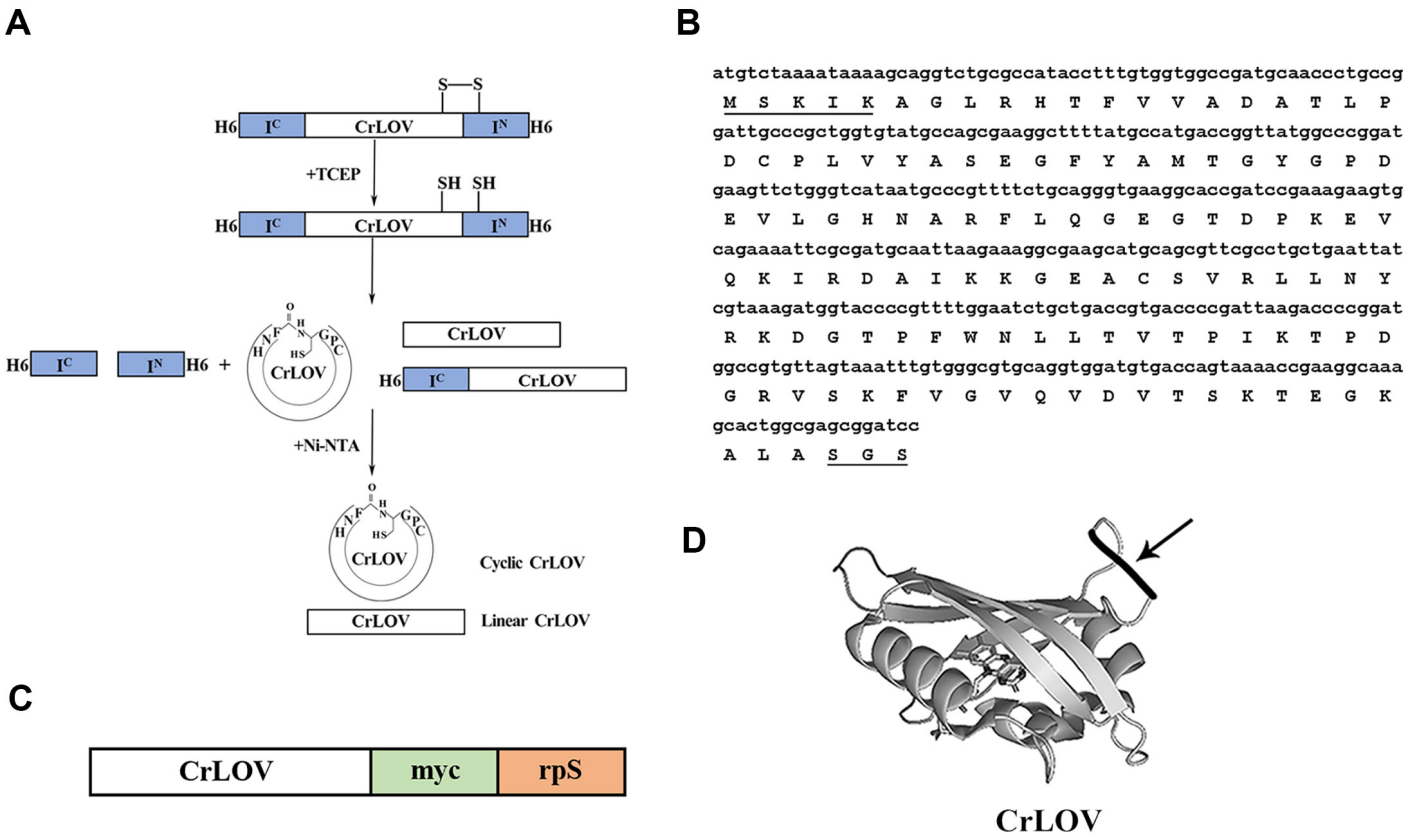
Use of the split intein construct for cyclization of the CrLOV. (**A**) Schematic presentation of the circular permutation approach for backbone cyclization of the CrLOV via TCEP-dependent protein trans-splicing reaction. (**B**) The gene encoding the CrLOV codon variant is presented. The extra amino acids as the linker are indicated by underline. (**C**) The CrLOV1 with the C-terminal myc tag and rpS for cyclization followed by linearization with purified R3P proteolysis. (**D**) Model of the cyclic CrLOV from the crystal structure (PDB code: 1N9L) is drawn by the PyMOL program (https://pymol.org). The linker in the cyclized CrLOV is indicated by the arrow (not to scale).

The codon-optimized CrLOV was incorporated into the split intein to generate the so-called sandwich protein. Under an oxidized environment, a disulfide bond is theoretically formed in the generated CPGC motif to retard the cyclization reaction in vivo. After the double His6-tagged precursor protein was purified by Ni-NTA matrix, the disulfide bond was reduced by addition of the reducing reagent, and the CrLOV was cyclized in vitro (Fig. 1A). The precursor protein, the C- domain (I^C^) of the Npu DnaE intein fused to the CrLOV, and the circularly permutated two-domain fusion were bound to Ni-NTA resin, whereas the linear protein as the side product and c-LOV were eluted from the affinity resin with buffer A. The sequences encoding the codon-optimized CrLOV are presented, and the linker was incorporated to ensure the correct conformation after cyclization (Fig. 1B). The cyclic CrLOV contains the linker CFNHMDKIKSGSCPG. The cyclized CrLOV1 contains the linker CFNHMEQKLISEEDLRSLEVLEQGPGSCPG composed of the myc-tag and rpS (Fig. 1C), for detection of the purified CrLOV1 with western blotting, and the different shifts of the cyclized and linearized CrLOV1 on the SDS-PAGE. Based on the crystal structure, the linker for c-LOV was indicated (Fig. 1D).

### Detection of the Precursor Protein Production and In Vivo Cyclization

**Fig. 2:**
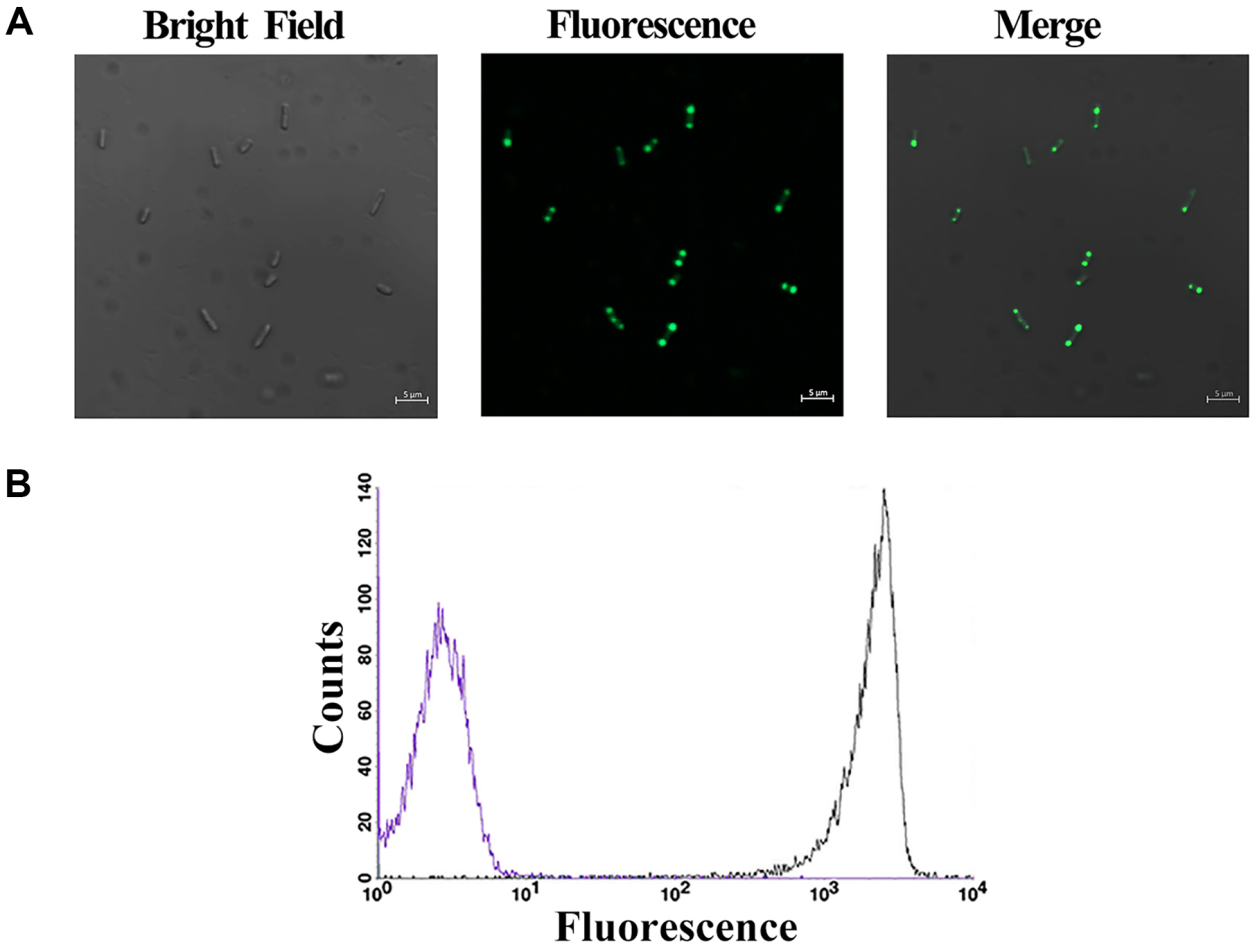
Fluorescence microscopic detection and flow cytometric analysis. (**A**) Microscopic image of SHuffle T7 cells (left), fluorescence from the coexpression system (middle), and the merged cells (right). (**B**) Flow cytometric analysis of the CrLOV fluorescence. The background fluorescence from cells carrying the p28Npu plasmids is designated in purple line, and the fluorescence from cells overexpressing the precursor protein for cyclizing the CrLOV is indicated in black line.

SHuffle T7 cells overexpressing the precursor protein with the hPDI were cultured aerobically. Under fluorescence microscopy, cells emitted fluorescence. Unexpectedly, localized distribution of the fluorescence was clearly detected (Fig. 2A). In contrast, cells overexpressing the precursor for cyclizing the EmGFP exhibit uniform green fluorescence throughout the cytoplasm [20]. The cell populations were distinguished by the flow cytometer (Fig. 2B). The results suggested that the CrLOV reporter was less efficient than the EmGFP in *E. coli* SHuffle T7 cells cultured aerobically.

### Purification of Two Types of LOVs

**Fig. 3:**
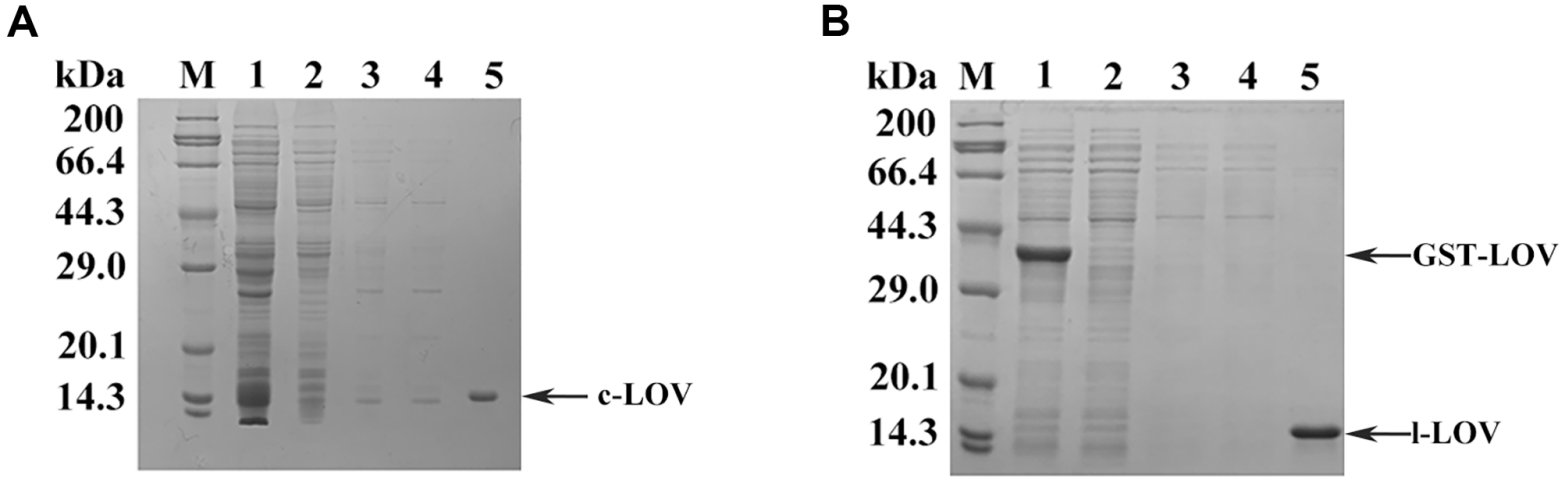
Purification of c-LOV and l-LOV. (**A**) SDS-PAGE analysis of the purified cLOV. M: protein marker. 1: the supernatant from SHuffle T7 coexpression system. 2: flow-through of the supernatant from Ni-NTA matrix. 3: eluted proteins with buffer B. 4: eluted proteins with buffer B containing 30 mM imidazole (pH 8.0). 5: the c-LOV was eluted with buffer B containing 30 mM imidazole (pH 8.0). (**B**) SDS-PAGE analysis of purified l-LOV produced in *E. coli* BL21 (DE3). 1: the induced GST-CrLOV. 2: Flow-through from Ni-NTA resin. 3: impurities washed with buffer B. 4: impurities washed with buffer B containing 30 mM imidazole (pH 8.0). 5: the released tag-free CrLOV as the l-LOV after on-resin cleavage.

As shown on the SDS-PAGE gel, a faint band close to the main band was observed on the SDS-PAGE gel from the eluent for purifying c-LOV (Fig. 3A). Certain split intein constructs catalyze protein circularization in vivo and in vitro. With the intein self-cleavage as the side reaction, the linear proteins are generated, and display slower shift than the cyclized proteins on the SDS-PAGE gel [16, 18, 20]. The l-LOV was also purified by using on-column cleavage of tevS in the His6-GST tagged CrLOV bound to Ni-NTA resin, and displayed one band on the SDS-PAGE gel (Fig. 3B).

### Fluorescence Spectra of Two CrLOV Species

**Fig. 4:**
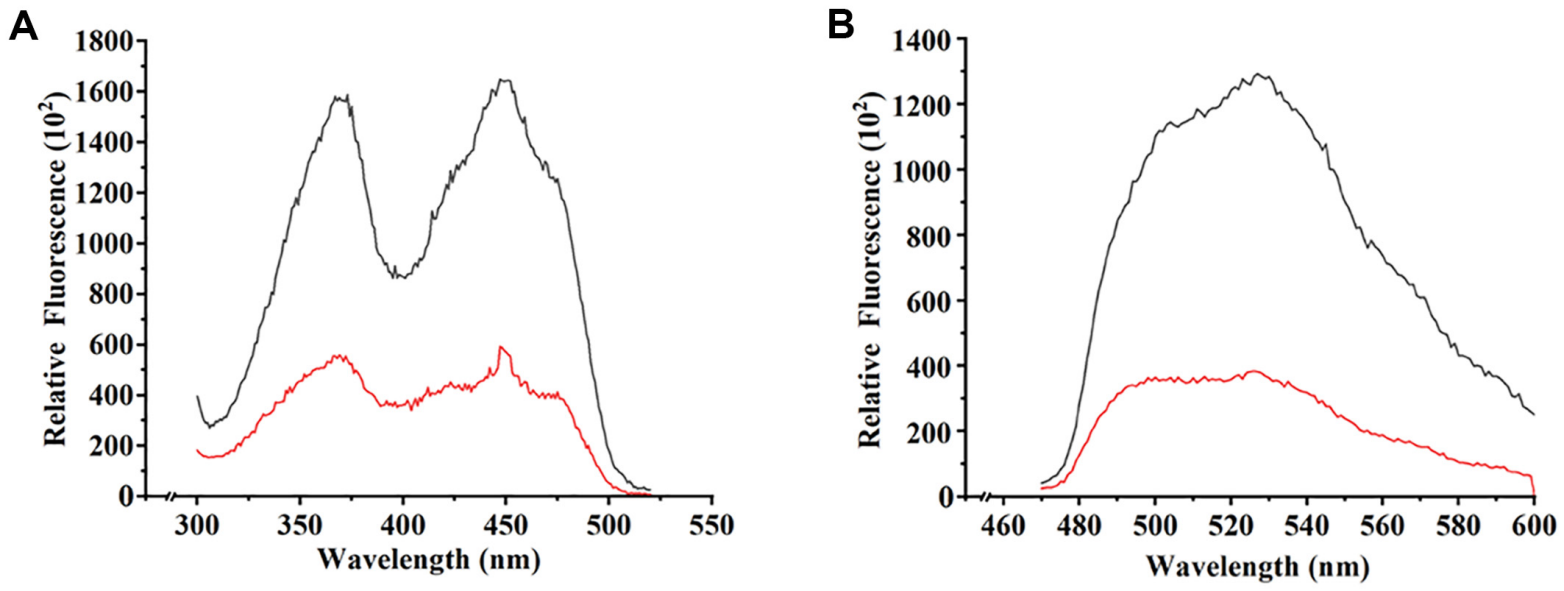
Fluorescence spectra of c-LOV and l-LOV. (**A**) Excitation spectra. (**B**) Emission spectra. Two protein species at the same concentration were analyzed. The spectra from the c-LOV were indicated in black lines, and those from l-LOV were designated in red lines.

Two CrLOV species purified under the same concentrations exhibited peaks at regions around 370 nm and 450 nm of the excitation spectra (Fig. 4A). The c-LOV with excitation at 370 nm and 450 nm was increased by about 3.2 folds, compared with the l-LOV (Fig. 4A). Both protein species displayed emission spectra with shoulders at 525 nm (Fig. 4B). The emission peak at 525 nm from c-LOV was about 3.2 folds higher than that from l-LOV. The results indicated that backbone cyclization obviously increased the LOV fluorescence.

### Analysis of the Cyclized CrLOV1 with the Relatively Long Linker

**Fig. 5:**
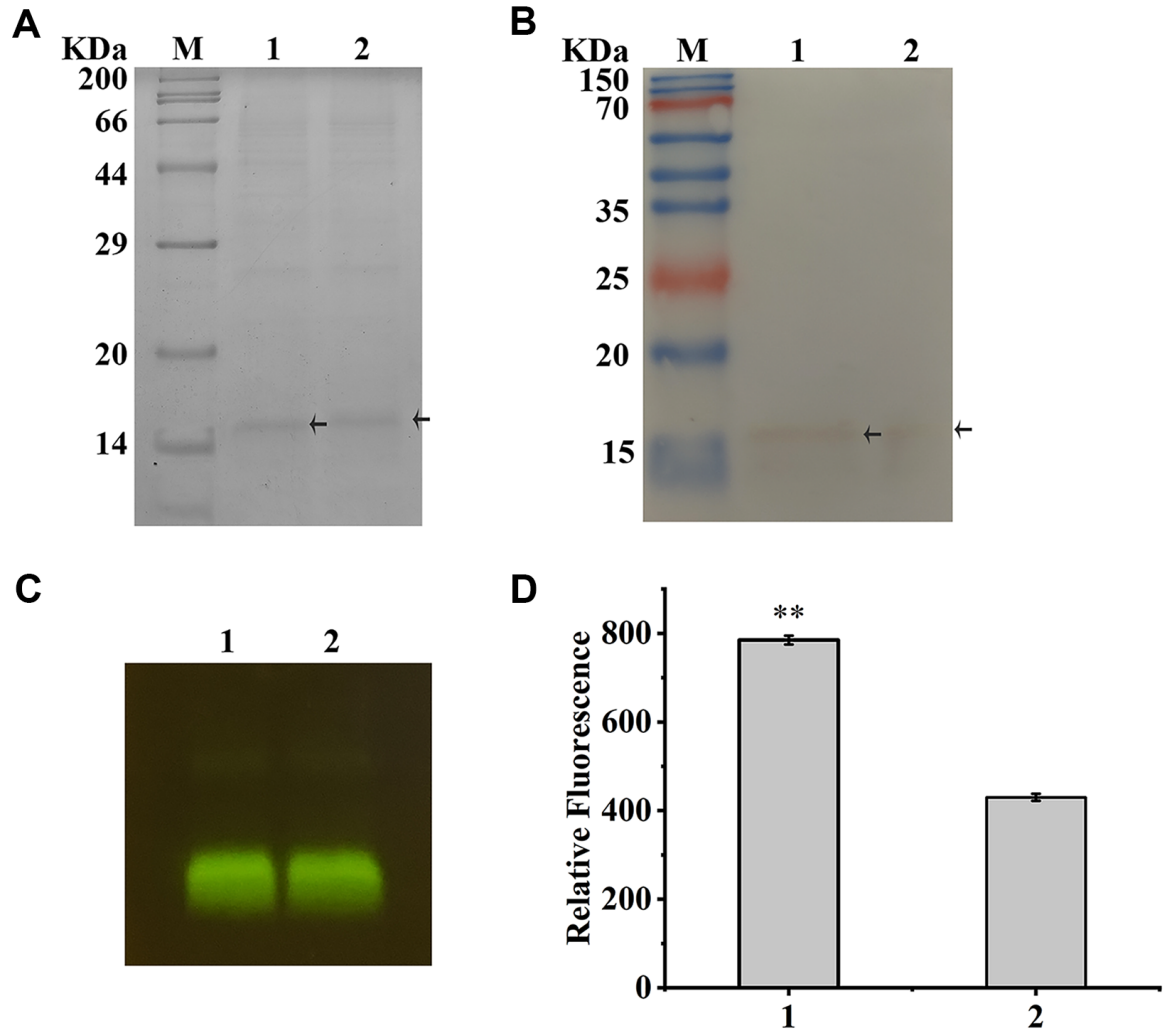
Linearization of the circular CrLOV1 by the purified R3P digestion. (**A**) SDS-PAGE analysis. The cyclized CrLOV1 protein samples without or with the R3P incubation were indicated as “1” and “2”. The circularized and linearized CrLOV1 were indicated by arrows. (**B**) Western blot analysis of the correspondent protein samples. (**C**) Fluorescence from the protein samples on the SDS-PAGE gel. (**D**) Relative fluorescence of the correspondent samples.

The l-LOV was released from the GST-LOV. Because the folding efficiency of 1-LOV was different from that of c-LOV, we incorporated CrLOV1 into the split intein for in vitro cyclization and linearization. The rpS was introduced into the C-terminus of CrLOV1. The purified R3P fusion showed high purity (Fig. S1A), and it efficiently cleaved the overexpressed GST-rpS-EmGFP (Fig. S1B). Incorporation of the myc tag and rpS sequence caused certain impurities to be co-eluted with the cyclic protein during purification, as detected by SDS-PAGE in a 15% polyacrylamide gel. Moreover, the protein linearized with the R3P displayed slightly slower migration than the cyclized protein (Fig. 5A). Western blot analysis showed the band appeared for the cyclized or linearized protein (Fig. 5B), but the shift difference on the gel for western blotting was not obvious, due to low protein amounts. The cyclic and linear small ubiquitin-like modifier (SUMO)-containing constructs are generated using the split intein for in vivo cyclization of the SUMO construct with a molecular mass close to that of CrLOV1, and the two protein species at relatively high amounts in crude extracts showed an observable shift difference on a 13%polyacrylamide gel [13].

The yield of cyclized CrLOV1 after purification and ultrafiltration was about 0.5 mg/L culture, whereas that of c-LOV was about 3.8 mg from 1 L culture. Incorporation of the tevS decreases solubility of several proteins [23], but the GST-tevS-GFP emitted fluorescent *E. coli* SHuffle T7 cells brighter than the GST-rpS-GFP (Fig. S2A), and fluorescence intensities from cells overexpressing the GST-rpS-GFP were about half of those from cells producing the GST-tevS-GFP (Fig. S2B). The results indicated that the rpS incorporation on decreasing protein solubility and folding was more effective than the tevS incorporation. The fluorescent band shows almost identical mobility on the SDS-PAGE gel (Fig. 5C), owing to the protein samples incubated with the loading buffer at 37°C for 10 min. The relative fluorescence of the cyclized CrLOV1 was about 1.8 folds higher than that of the linearized one (Fig. 5D), suggesting that the relatively long linker designed in this work decreased the cyclization effect on enhancing the CrLOV fluorescence.

### Thermostability of Two CrLOV Species

**Fig. 6:**
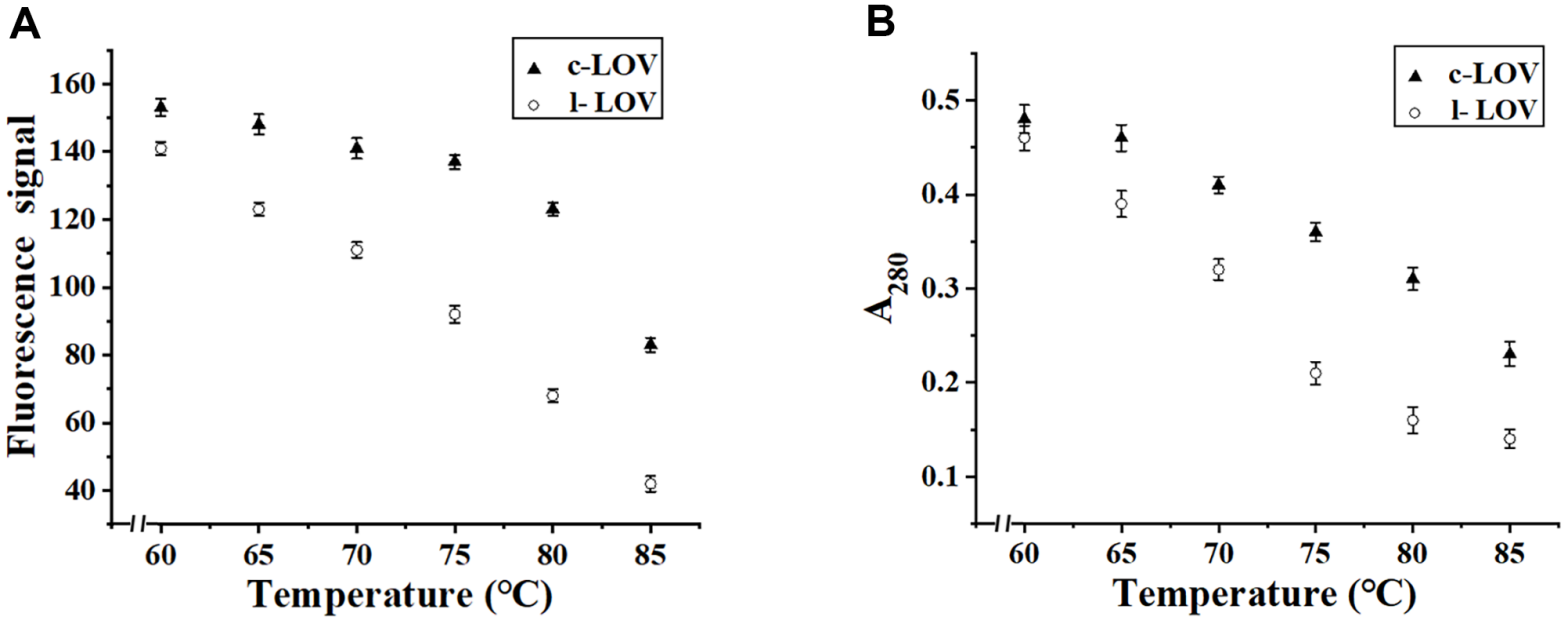
Thermostability of the purified c-LOV and l-LOV. (**A**) Fluorescence signals from soluble c-LOV and l-LOV after proteins were incubated for 10 min at different temperatures and centrifuged. (**B**) A_280_ values representing soluble amounts of samples after heat treatment.

Because the c-LOV fluorescence was stronger than that of the cyclized CrLOV1, we selected c-LOV for examination of the thermostability. At a temperature regime of 60–85°C with 5°C increments, fluorescence decrease in c-LOV was less abrupt than that in l-LOV (Fig. 6A). The initial fluorescence was retained at about 30% in the purified l-LOV, but roughly 60% in the purified c-LOV. When the temperature was increased, c-LOV showed less insoluble aggregation than l-LOV, based on the A_280_ measurement of soluble fractions (Fig. 6B). Thus, the sharper fluorescence decrease in l-LOV was attributed to the thermal denaturation of the purified proteins. After heating, two soluble protein species were analyzed by SDS-PAGE (Fig. S3A, S3B). At 80°C incubation for 10 min, most amounts of c-DLA were higher than those of l-LOV. The fluorescence from the treated c-LOV and l-LOV was detected on SDS-PAGE gel (Fig. S3C, S3D). This analytical method was less sensitive for assessing the thermostability of CrLOV.

### Sensitivity of Two CrLOV Species to CPY Digestion

**Fig. 7:**
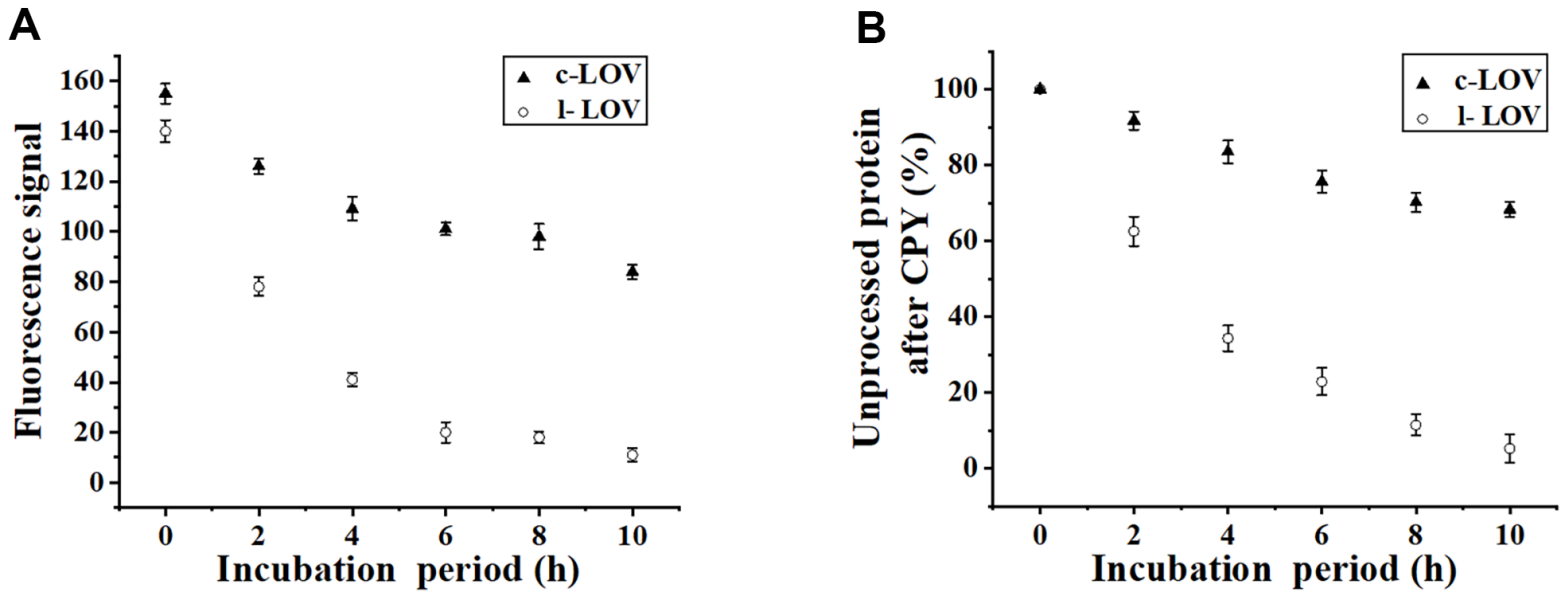
The CPY mediating the limited proteolysis of c-LOV and l-LOV. (**A**) Fluorescence signals from two CrLOV species with proteolytic treatment. (**B**) The retained intact protein ratio of two CrLOV species after CPY incubation.

With the increased CPY incubation periods, the fluorescence from purified c-LOV was slightly decreased (Fig. 7A), since the purified c-LOV contained the linear CrLOV. In contrast, the abrupt decline in fluorescence from purified l-LOV treated with CPY was identified (Fig. 7A). Based on the estimated intact protein amounts, l-LOV was degraded more easily than the c-LOV (Fig. 7B). SDS-PAGE analysis (Fig. S4A, S4B) and fluorescence display on SDS-PAGE gel (Fig. S4C, S4D) showed that purified c-LOV was more tolerant to CPY than purified l-LOV.

### Fluorescence from Cells Overexpressing the CrLOV Constructs in Oxygen-Deficient Culture

**Fig. 8:**
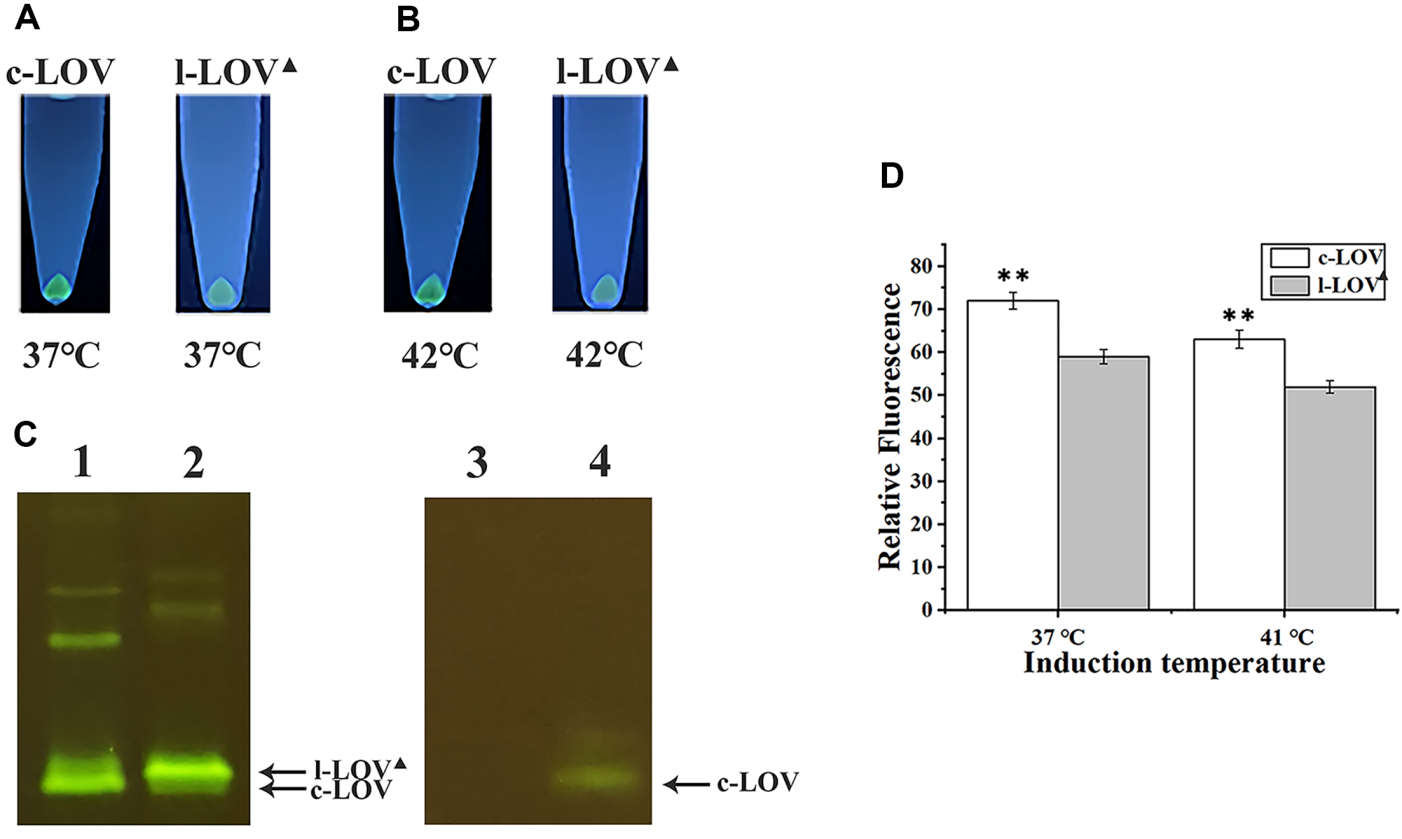
Functional production of the precursor for in vivo cyclization of the CrLOV, and the linear CrLOV construct. *E. coli* BL21(DE3) cells were cultivated in sealed tube with full of LB culture during protein induction at two specified temperatures. (**A**) Cells were induced at 37°C. (**B**) Cells were induced at 41°C. After induced, cells were collected, exposed to the UV light and photographed. The SFNH residues at N-terminus of the l-LOV were deleted in the l-LOV▲, as indicated in the figures. (**C**) Fluorescent bands were displayed on SDS-PAGE gels for detecting the target proteins induced at 37°C in the absence (left) or presence (right) of the CPY digestion. 1 and 2: clear lysate for overexpressing the precursor and l- LOV▲. 3 and 4: CPY digestion of the correspondent protein samples with dilution to 10 folds. The c-LOV and l-LOV▲ were indicated by arrows. (**D**) Relative fluorescence from cells induced at the specified temperatures. The asterisk indicates significant differences higher than the control; *p < 0.01. The experiments were described in the section of “Materials and Methods”.

To create an oxygen-deficient environment, cells were grown in a sealed tube full of the culture to exhaust the limited oxygen. Two CrLOV constructs in BL21(DE3) cells in a sealed Eppendorf tube were individually induced at 37°C and 41°C, respectively. Fluorescent cells producing precursor proteins after collection were observed, and were brighter than cells overexpressing the linear CrLOV construct (Fig. 8A, 8B). The oligomers of the linear CrLOV construct in soluble fraction were identified (Fig. 8C), due to the protein sample with SDS-PAGE loading buffer incubated at 37°C for 10 min, with loaded proteins in high amounts. The oligomerized red fluorescent protein amounts were detected based on fluorescent bands on the SDS-PAGE gel [13]. When the crude extracts were diluted to 10-fold, and incubated with CPY for 24 h, fluorescence emitted from c-LOV on the gel was observed, whereas the linear CrLOV was degraded, leading to the disappearing of fluorescence (Fig. 8C). The fluorescence intensities of cells overexpressing the precursor were higher than those producing the linear CrLOV construct (Fig. 8D). The results suggested that c-LOV played a greater role in cell fluorescence than the linear CrLOV construct.

To further determine the limited-oxygen environment in the sealed tube, we used the oxygen-dependent EmGFP reporter. *E. coli* BL21(DE3) cells overexpressing the EmGFP cultured aerobically were fluorescent under UV irradiation, but those induced under hypoxic conditions were weakly fluorescent (Fig. S4A), most likely attributed to leaky expression of the EmGFP, and the presence of micro-oxygen in the sealed tube. The fluorescence intensity of induced cells under aerobic cultivation was significantly higher than that under anoxic condition (Fig. S4B). The OD_600_ value for cells in aerobic culture was higher than that in the oxygen-poor culture (Fig. S4C). The expression level of the EmGFP cultured aerobically was slightly higher than that cultured in a low concentration of oxygen, as detected by western blotting (Fig. S4D). The current findings suggested that c-LOV was functional in vivo at a raised temperature in anoxic conditions. Induction of the fluorescent protein in a sealed tube full of culture is conducted as a way to simply and easily detect a reporter dependent on or independent of oxygen.

## Discussion

As in our previous report [20], the developed split intein system was also efficient for CrLOV circularization. After induction, fluorescence in the particulate morphology of the bacteria cell overexpressing the constructed CrLOV aerobically was identified under fluorescence microscope (Fig. 2A), different from the homogenous fluorescent cells overexpressing the LOV-based reporters [24, 25], but similar to other observations [26, 27]. As a comparison, the fluorescence from the precursor for cyclizing EmGFP is uniform in cells [20]. The non-uniform fluorescence emitted from CrLOV in this work was possibly the result of CrLOV photobleaching, and a limited endogenous level of FMN. We confirmed that the photoswitchable LOV domain causes the confined fluorescence in *E. coli* cells [24]. The photobleaching time constant in the ﬁxed cells overexpressing the CrLOV was about 26 s [5].

The cyclic and linear CrLOV proteins displayed comparable excitation peaks at about 370 nm and about 450 nm (Fig. 4A), similar to the free FMN spectra [28]. In contrast, excitation at 450 nm for the purified His6-tagged CrLOV was higher than that at 340 nm [5]. However, we exchanged buffer when dissolving either of the CrLOV species by ultrafiltration. Thus, free FMN was not present in the purified CrLOV solution. The emission peak of free FMN was about 530 nm [28], faintly red-shifted to that of the purified CrLOV, as measured in this work, as well as in another previous report [5]. The excitation peak difference was probably attributed to photobleaching of the partial CrLOV, due to approximately 34 s photobleaching time constant in vitro [5].

Backbone cyclization of the fusion proteins was determined by electrospray ionization-tandem mass spectrometry, which requires highly pure proteins [18]. Fusion of the C-terminal His6-tag to the target protein, and CPY proteolysis of the reaction mixture followed by immobilized metal ion affinity chromatography was used to identify the cyclization of the target protein [29]. In this and previous studies, we independently constructed and purified cyclic and linear protein reporters, and compared the hydrolytic stability. Recently, linear and cyclic trimers of *Staphylococcus aureus* protein A domain Z were also constructed and purified, and cyclic protein with MALDI-TOF mass identification was resistant to CPY proteolysis [30]. The cyclic protein migrating faster than the linearized one on SDS-PAGE gel is a simple analytic approach to identify protein circularization. For example, thrombin mediates linearization of the cyclized GFP with the introduced LVPRG, and the linearized GFP migrates more slowly than the cyclized one [16, 17, 31]. In this work, we introduced the rpS to identify the backbone cyclization using purified R3P for linearizing the cyclic protein, by observation of the differential shift on SDS-PAGE gel. However, the rpS decreased protein solubility.

The fluorescence emitted from the LOV domain is dependent on the bound FMN. The crystal structure of LOV is dissimilar to that of the GFP as the β barrel (Fig. 1C). Lack of C-terminal Ja helix of Arabidopsis LOV domain leads to an enhanced tendency to form inclusion bodies produced in *E. coli* [32]. Crystal structures reveal that the Ja helix contributes to an increase in LOV domains’ overall surface hydrophilicity [33, 34]. Cyclization most likely limits mobility of the Ja helix of the CrLOV, but the cyclization effect on enhancing CrLOV fluorescence was related to the length and constitution of the incorporated linker.

Cell fluorescence was measured under two cultural conditions in the presence of different oxygen levels. In cells overexpressing the precursor for in vivo cyclization, the unspliced precursor protein, the I^C^-CrLOV by-product, the linear and cyclic CrLOVs contributed to cell fluorescence. The I^C^ is not codon-optimized, and it displays little impact on enhancing the EmGFP productivity for cyclization in the precursor, as compared to the tag-free EmGFP [20]. In this work, the linear CrLOV construct for detecting protein fluorescence in a simulated anaerobic environment contains the N-terminal SKIK tag encoded by the designed codons. This tag is effective for enhancing protein productivity in *E. coli* [35]. The expression level of the linear LOV is reasonably higher than that of the precursor. Therefore, the increased fluorescence of cells overexpressing the precursor in the oxygen-poor culture probably resulted from the c-LOV contribution.

The superfolder GFP variant is fluorescent in thermophilic aerobic bacteria [36]. The fluorescent protein reporter used in thermophilic anaerobic bacteria is still lacking, and c-LOV is expected to be a useful tool. It is plausible that the thermostable split Cfa intein engineered from the Npu DnaE one [37], combined with the thermostable LOV selected from the thermophilic bacteria, enables the cyclized LOV domain to function better in extremely thermophilic anaerobic bacteria through genetic transformation. Even in the absence of the redox trap, the split Npu DnaE is sensitive to the oxidized environment [38]. Accordingly, the cyclization activity is increased for cells overexpressing the precursor protein cultured anaerobically.

## Conclusion

This work presented the use of disulﬁde-trapped intein technology for the production of two cyclized CrLOV constructs with different linkers. Compared with l-LOV, c-LOV displayed enhanced fluorescence, thermal stability, and resistance to exopeptidase proteolysis. Under low-oxygen cultivation during protein induction, *E. coli* cells overexpressing the precursor for CrLOV circularization in vivo displayed stronger fluorescence than those producing the non-tagged CrLOV, thereby providing a stable c-LOV alternate for potential use in certain thermophilic anaerobic bacteria species.

## Supplemental Materials

Supplementary data for this paper are available on-line only at http://jmb.or.kr.
