# Supplementary material for: Backbone Cyclization of Flavin Mononucleotide-Based Fluorescent Protein Increases Fluorescence and Stability
Source: J Microbiol Biotechnol. 2023 Aug 28;33(12):1681–91. doi: 10.4014/jmb.2305.05011 (PMC10772547; doi:10.4014/jmb.2305.05011)
Supplement: Supplementary file 1 [file jmb-33-12-1681-supple.pdf]

Supplementary Table and Figures

Backbone Cyclization of Flavin Mononucleotide-Based Fluorescent Protein Increases Fluorescence and Stability

Tingting Lin, Yuanyuan Ge, Qing Gao, Di Zhang, Xiaofeng Chen, Yafang Hu, Jun Fan*

Table S1 Primers used in this study

Primer	Sequence(5'-3')
P1	F: GTACTACATATGTCTAAAATAAAAGCAGGT
P2	R: CATGGATCCGCTCGCCAGCGCTTTGCCTTC
P3	F: CTAAGATCTTTCAACCATATGGATAAAATAAAAG
P4	R: CTACTCGAGTTAGCCAGGGCAGGATCCGCTC
P5	R: CACTAATCAGTTTCTGTTCTGCCAGTGCTTTGCCTTC
P6	R: GATAGATCTCAGGTCTTCTTCACTAATCAGTTTCTG

F: forward primer; R: reverse primer. The cut sites of the restriction endonucleases are indicated by underlines.

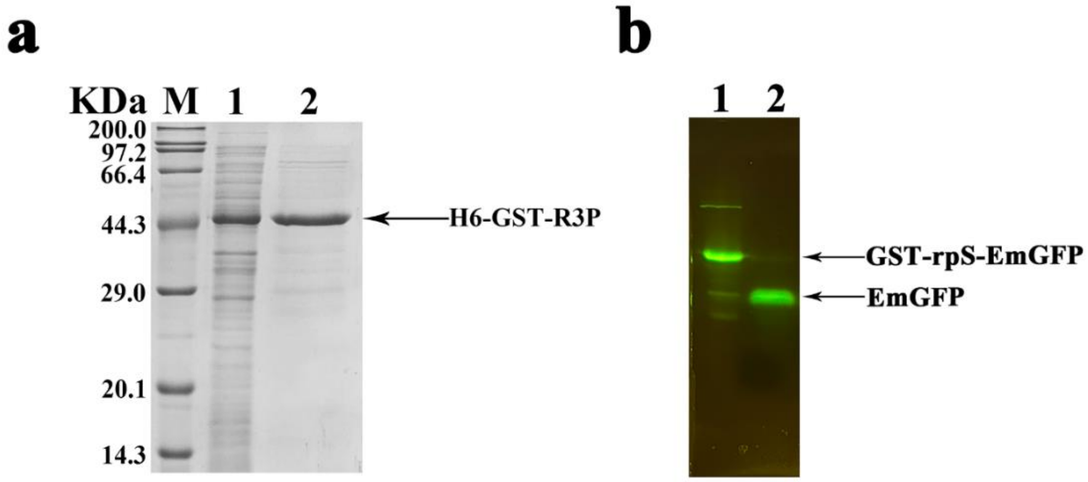

Fig. S1 Purification and activity assay of the His6-GST tagged R3P. a SDS-PAGE analysis of the purified R3P partner. 1: the overexpressed target protein in clear lysate. 2: the fusion protein eluted from Ni-NTA agarose. Arrow indicates the fusion protein for the R3P. **b** Fluorescent band on the SDS-PAGE gel was displayed after the fusion construct containing the R3P cleavage sequence rpS was cleaved in vitro. The fusion protein and the released tag-free EmGFP are indicated by arrows.

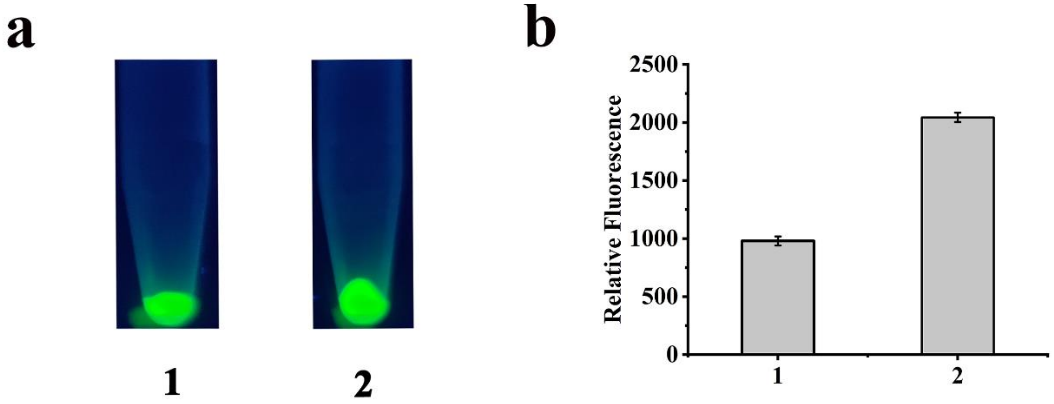

Fig. S2 Cell fluorescence from the overexpressed fusion proteins is used to compare the incorporated cleavable sequences affecting protein solubility. a The collected cells overexpressing GST-rpS-EmGFP and the GST-tevS-EmGFP were indicated as “1” and “2”, exposed under UV light, and photographed. **b** Relative fluorescence of the correspondent samples.

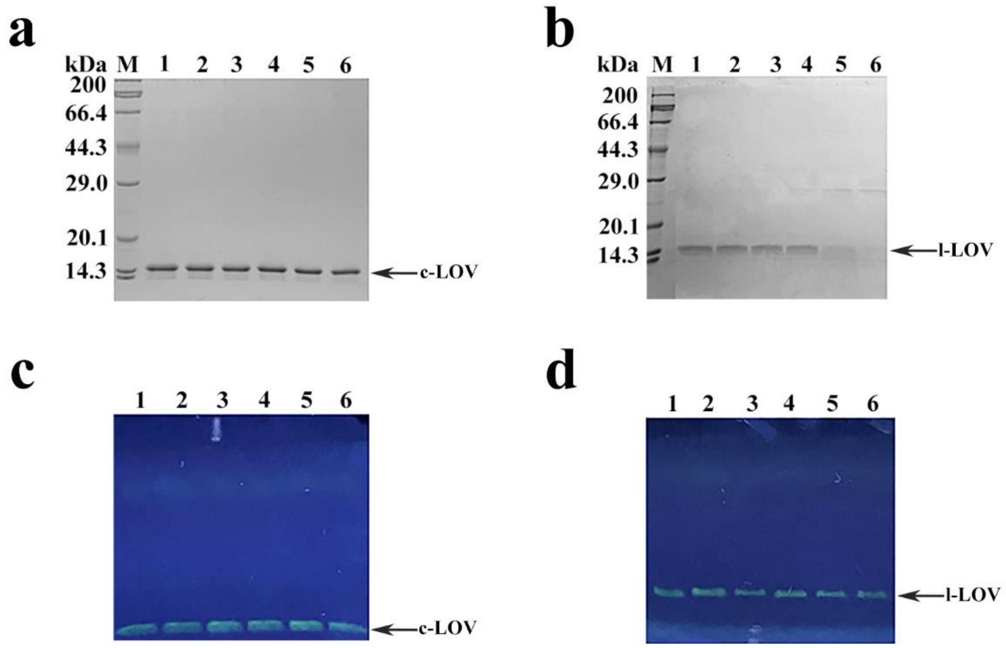

Fig. S3 Analysis of purified c-LOV and l-LOV with heating treatment. a and **b** Almost equal amounts of the two type of proteins at initial treatment (at 60 °C) were used as the standard for further investigating the thermostability. In each figure, lanes 1 to 6 indicated that proteins were heated for 10 min between 60~85 ℃ for every 5 ℃ increment, centrifuged and soluble fractions were analyzed. **c** and **d** The fluorescence display of the correspondent protein samples heated at the specified temperatures.

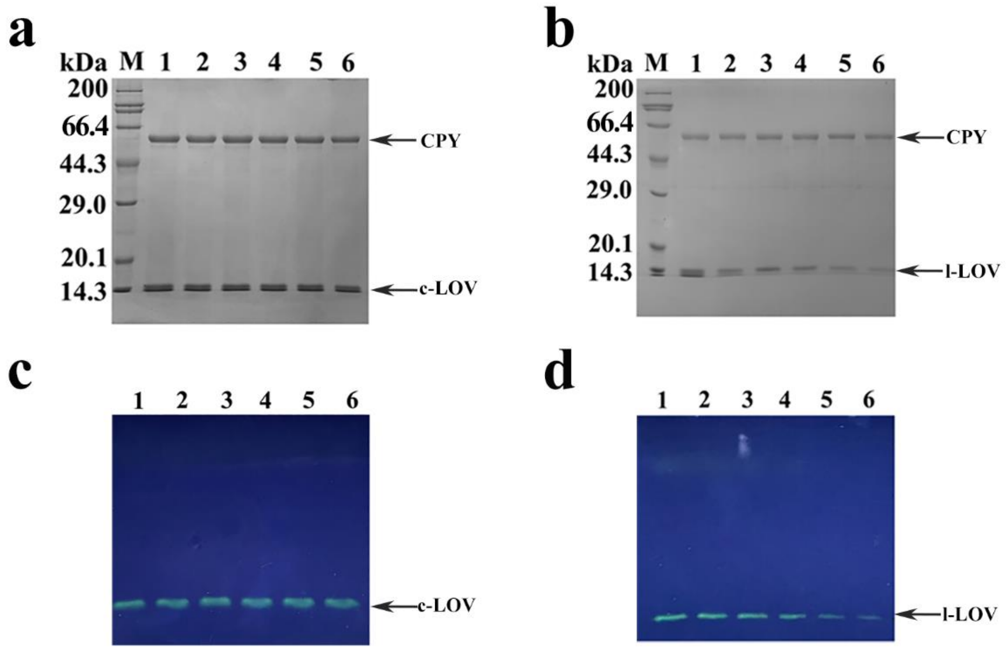

Fig. S4 Analysis of purified c-LOV and l-LOV with CPY treatment. a and **b** Almost equal amounts of the two type of proteins upon addition of CPY without incubation were used as the standard for further investigating the hydrolytic stability. In each figure, lanes 1 to 6 indicated that proteins were incubated with CPY from 0 to 10 h for every 2 h increment at 25 ℃. **c** and **d** The fluorescence display of the correspondent protein samples with CPY incubation at different periods.

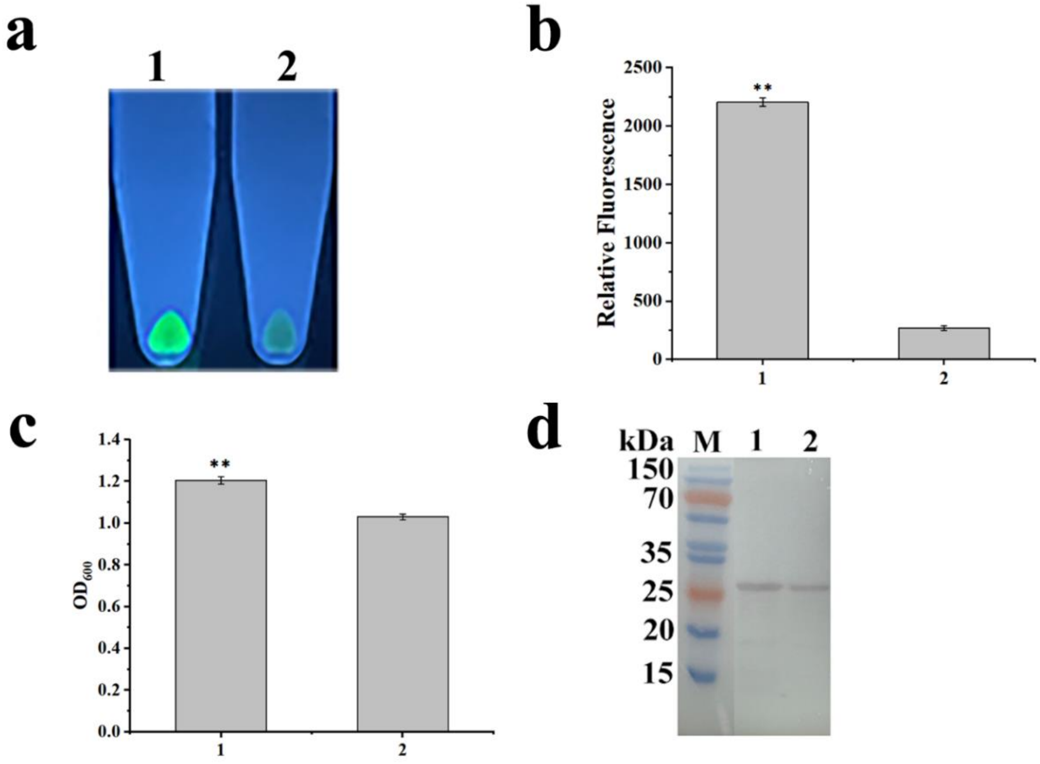

Fig. S5 Production of the His6-tagged EmGFP in BL21(DE3) cells in oxygen dependent and independent conditions. a Fluorescence display of the collected cells for inducing protein at 37 °C. 1: cells cultured aerobically during protein induction. 2: cells cultured in oxygen-deficient environment during protein induction. **b** Relative Fluorescence intensities from induced cells cultured under two conditions, respectively. **c** Cell densities from the correspondent samples. **d** Western blot analysis of the His6-EmGFP using anti-His6 monoclonal antibodies. The asterisk indicates significant differences higher than the inactive precursor as a control; * $p < 0.01$. The experiments were described in the section of “Materials and Methods”.
